# The feasibility of whole body vibration in institutionalised elderly persons and its influence on muscle performance, balance and mobility: a randomised controlled trial [ISRCTN62535013]

**DOI:** 10.1186/1471-2318-5-17

**Published:** 2005-12-22

**Authors:** Ivan Bautmans, Ellen Van Hees, Jean-Claude Lemper, Tony Mets

**Affiliations:** 1 Gerontology, Free University of Brussels (VUB), Laarbeeklaan 103, B-1090 Brussels, Belgium; 2 Revalidation Sciences & Physical Therapy, Free University of Brussels (VUB), Laarbeeklaan 103, B-1090 Brussels, Belgium; 3 Physical Therapy, Hogeschool Antwerpen, Antwerp, Belgium; 4 Geriatrics, Academic Hospital of the Free University of Brussels (AZ-VUB), Laarbeeklaan 101, B-1090 Brussels, Belgium; 5 Foundation for Psychogeriatrics, Brussels, Belgium

## Abstract

**Background:**

Fatigue or lack of interest can reduce the feasibility of intensive physical exercise in nursing home residents. Low-volume exercise interventions with similar training effects might be an alternative. The aim of this randomised controlled trial was to investigate the feasibility of Whole Body Vibration (WBV) in institutionalised elderly, and its impact on functional capacity and muscle performance.

**Methods:**

Twenty-four nursing home residents (15 female, 9 male; mean age 77.5 ± 11.0 years) were randomised (stratification for age, gender and ADL-category) to 6 weeks static WBV exercise (WBV+, N = 13) or control (only static exercise; N = 11). Outcome measures were exercise compliance, timed up-and-go, Tinetti-test, back scratch, chair sit-and-reach, handgrip strength and linear isokinetic leg extension.

**Results:**

At baseline, WBV+ and control groups were similar for all outcome variables. Twenty-one participants completed the program and attended respectively 96% and 86% of the exercise sessions for the WBV+ and control groups. Training-induced changes in timed up-and-go and Tinetti-test were better for WBV+ compared to control (p = 0.029 for timed up-and-go, p = 0.001 and p = 0.002 for Tinetti body balance and total score respectively). In an alternative analysis (Worst Rank Score & Last Observation Carried Forward) the differences in change remained significant on the Tinetti body balance and total score. No other significant differences in change between both groups were observed.

**Conclusion:**

In nursing home residents with limited functional dependency, six weeks static WBV exercise is feasible, and is beneficial for balance and mobility. The supplementary benefit of WBV on muscle performance compared to classic exercise remains to be explored further.

## Background

In old age, muscle weakness due to sarcopenia is responsible for the development of frailty and important disability [1-3]. Especially in institutionalised elderly persons, muscle strength can deteriorate to a point where it becomes critical for independence of transfers and walking.

There is strong evidence that in healthy older persons major gains in muscle strength can be obtained by resistance exercises [4]. Also in frail institutionalised elderly resistance training is feasible [5,6], and can lead to clinically relevant strength gain and improved mobility [7,8].

However, intensive resistance training programmes targeting key muscle groups necessary for transfers and walking can attain considerably high exercise volumes, representing a combination of total exercise duration (including rest periods), intensity (70–80% of maximal resistance) and number of repetitions (3 series of 10 repetitions). Consequently, fatigue, a symptom reported by 98% residents of a long-term care facility [9], or lack of motivation can reduce its feasibility in frail elderly subjects.

Whole body vibration (WBV) is a new training method using an oscillating platform upon which exercises are performed (e.g., standing, static or dynamic). A small volume of this type of exercises has been reported to lead to significantly improved muscle function in young [10,11] and healthy elderly persons [12].

The aim of this randomised controlled trial was to investigate the feasibility of WBV in frail institutionalised elderly persons, and its impact on muscle performance and functional capacity.

## Methods

### Participants

All residents of a nursing home (Van Zanden, Brussels, Belgium; capacity of 102 beds) within dependence categories O, A and B according to the scale of Katz et al. [13] for basic activities of daily living (ADL) were eligible. Exclusion criteria were mainly based on contra-indications for WBV: presence of infectious disease, insulin-dependent diabetes mellitus, endogenous osteosynthethical material, knee or hip prosthesis, pacemaker, epilepsy, musculo-skeletal disorders and cognitive or physical dysfunction interfering with test and training procedures.

At the moment of the study, 98 subjects (74 female and 24 male) resided in the nursing home, among whom 62 were eligible (N = 39 and N = 23 for category O/A and B respectively). Thirty-Three persons showed no exclusion criteria, among whom 24 gave informed consent to participate in the study (15 female and 9 male, mean age 77.5 ± 11.0 years). All participants were naïve for WBV. The local ethical committee approved the study.

### Randomisation

Randomisation was done for all 24 participants together at the same moment by lottery, thus eliminating selection bias, that can occur with sequential enrolment [14]. One group was assigned to 6 weeks static WBV exercise (WBV+) and another group to the same exercise regimen without WBV (control). Stratification was applied for gender, dependence for ADL, and age. A-priori, we considered subjects in ADL-category O or A possibly different from those in ADL-category B; and the oldest old possibly different from younger ones (cut-off at age 84 years). Exact distribution of 12/12 in intervention and control group was not an a-priori criterion.

For each participant a card was made containing identification number, gender (male or female), dependency level (O/A or B) and age (old or oldest old). Next, the cards were put in different baskets dividing the population into 8 subgroups according to the stratification criteria. From each basket separately, alternatively cards were assigned to the intervention or control group by means of lottery. For each basket separately, the starting sequence for lottery was determined by tossing a coin. Finally, 13 participants were assigned to WBV+ and 11 to control

### Intervention

The participants assigned to the WBV+ group performed a 6-week exercise program on a vibration platform (Power-Plate, Badhoevedorp, The Netherlands), which was installed for the study purpose in the rehabilitation room of the nursing home. This device provides a vertical vibration with a frequency of 30–50 Hz and an excursion of 2–5 mm. Exercises were performed three times per week (with a minimum of 1-day rest in between) and consisted in 6 static exercises targeting lower limb muscles. The exercise volume and intensity were progressively increased according to the overload-principle (table 1).

**Table 1:** Progression of WBV exercise program over 6 weeks training.

	**Warming-Up**	**Training**				
		Exercise	Duration	Frequency (Hz)	Amplitude (mm)	Rest (sec) ^a^

Week 1	Exercise 1	2	3 × 30 sec	35	2	60
	2 × 30 sec each leg	3, 4	1 × 30 sec	35	2	60
Week 2	Amplitude: 2 mm	2	3 × 30 sec	35	2	30–60
	Frequency: 30 Hz	3, 4, 5, 6	1 × 30 sec	35	2	30–60
Week 3		2	3 × 45 sec	40	2	30–60
		3, 4, 5, 6	1 × 45 sec	40	2	30–60
Week 4		2	3 × 60 sec	40	2	30–60
		3, 4, 5, 6	1 × 60 sec	40	2	30–60
Week 5		2	3 × 45 sec	40	2	30–60
		3, 4	1 × 30 sec	30	5	30–60
		5, 6	1 × 45 sec	40	2	30–60
Week 6		2	3 × 45 sec	40	2	30–60
		3, 4	2 × 30 sec	35	5	30–60
		5, 6	1 × 45 sec	40	2	30–60

Exercise 1 = Lunge, Exercise 2 = Squat, Exercise 3 = Deep squat, Exercise 4 = Wide stance squat, Exercise 5 = Calves, Exercise 6 = Calves deep (see [30] for detailed description of the exercises). ^a ^Amount of rest between each series of exercise.

Subjects in the control group performed exactly the same exercise program on the vibration platform as the WBV+ group, but without vertical vibration. In fact, the sound of the motor of the vibration platform was reproduced by a tape recorder during each bout of exercise. Hence, all subjects were convinced that the vibration platform was functioning during the exercises and thus were blinded for group assignment.

All participants wore identical adjustable sandals during the exercise.

During the study period all participants continued to attend two-weekly seated gymnastic sessions together with other residents of the nursing home (not participating in the study), which were organised by independent physical therapists who were unaware of the group assignment of the participants. The gymnastic exercises were performed on a chair and targeted social interaction of the residents.

### Measurements

Outcome measures were feasibility (taking into account continuation of the exercise program and/or occurrence of complications related to the WBV) and improvement in functional performance due to the intervention (taking into account balance and gait, upper limb and lower body flexibility, maximal grip strength and closed chain bilateral leg extension).

The participants' attendance and occurrence of complications due to the WBV were recorded at each exercise session. Functional performance was assessed at baseline and after 6 weeks training. At baseline, height and weight were measured, and body mass index and total body muscle mass were estimated in all participants.

#### Functional performance

Maximal grip strength of the dominant hand was measured using a Martin vigorimeter device (Elmed, Addison, USA), as described previously [15].

Balance and gait were assessed using the timed up-and-go test [16] and Tinetti-test [17].

Upper limb and lower body flexibility were assessed using the back scratch and chair sit-and-reach test. The back scratch test consists in reaching behind the head with one hand and behind the back with the other hand towards the middle finger of both hands [18]. The score is expressed as the distance (in cm) between both middle fingers. During the chair sit-and-reach test the subject sits on the front edge of a chair and extends one leg straight out in front of the hip, with the foot in dorsal flexion and the heel resting on the floor and reaches as far as possible toward the toes [18]. The result of the test is expressed as the distance (in cm) between the fingers and foot. In both tests the scores were negative when the subject was unable to touch the foot or the middle finger and positive when overlap with foot or middle fingers was possible. Both tests were performed twice with the preferential leg or arms and the best score was registered [19].

Closed chain bilateral leg extension was evaluated using the Aristokin^® ^(Lode, Groningen, The Netherlands), a linear isokinetic multi-joint dynamometer. Power (W), force (N), work (J) and explosivity (N/sec) developed during the movement were measured at 40 and 60 cm per second, as described previously [19]. High single-session reproducibility (ICC 0.85–0.99) and high intra-observer reliability (ICC 0.67–0.94) over a 6-week period are described for this technique in a young population [20].

Functional performance assessment was done by a physical therapist who was unaware of the group assignment of the participants.

#### Body composition & anthropometry

The body mass index was calculated as weight (kg)/height (m) ^2^. Total body skeletal muscle mass was estimated as described previously [21] with the formula:

Muscle Mass (g) = Height × (0.0553 CTG^2 ^+ 0.0987 FG^2 ^+ 0.0331 CCG^2^) - 2445.

where height in cm, CTG = thigh circumference corrected for the front thigh skin fold thickness (cm), FG = uncorrected forearm circumference (cm), and CCG = calf circumference corrected for the medial calf skin fold thickness (cm). [22]

### Statistical analysis

Statistical analysis was performed using SPSS for Windows (release 12.0). Average values are given ± their standard deviation (SD). Since the number of participants was small non-parametric techniques (with exact testing) were used: Wilcoxon Signed Ranks Test and Mann-Whitney U Test for paired and unpaired comparisons respectively. Significance level was set at two-sided p < 0.05.

## Results

At baseline, no significant differences were found between the WBV+ and control groups for any of the observed variables (table 2 &3). Twenty-one of the 24 participants completed the 6-week exercise program and attended respectively 96% and 86% of the exercise sessions for the WBV+ and control groups. Three subjects of the WBV+ group were unavailable for end-evaluation: one female presented groin pain (without apparent lesions upon physical examination) after the first exercise sessions and one female became afraid to go to the rehabilitation room; both refused further participation; one male developed airway infection during the study (accompanied with severe decline in clinical condition and confined to bed rest making re-assessment impossible). Observations are missing completely at random (MCAR) or ignorable when the presence or absence of an observation occurs purely by chance [23], and thus the chance of missing is equal for participants in both intervention and control groups. When posttreatment observations are MCAR, then the analysis of the observed (nonmissing) data is unbiased [24]. It can be assumed that the occurrence of airway-infection over a six-week period is similar for both WBV+ and control groups. Therefore we consider the latter case as MCAR and thus ignorable in our analysis. Data-analysis was performed considering the end evaluations of the two female subjects (who refused further participation) as missing values as well as by including them using the worst rank score analysis (WRS) and the last observation carried forward approach (LOCF). Since these approaches cause distortion of the mean values [23], raw data are reported based on N = 10 in the WBV+ group (table 4).

**Table 2:** Participants' characteristics at baseline.

**Parameter**	**WBV+**		**Control N = 11**	**p^b^**	**p^c^**
				
	Initial Randomisation (N = 13)	Reassessed at 6 weeks (N = 10)			
**Gender **(M/F)	5/8	4/6	4/7	-	-
**Age **(years)	76.6 ± 11.8	76.3 ± 9.7	78.6 ± 10.4	.943	.849
**Weight **(kg)	63.5 ± 14.3	66.7 ± 13.8	63.2 ± 21.1	.898	.592
**Height **(m)	1.61 ± 0.12	1.63 ± 0.09	1.63 ± 0.09	.787	.987
**BMI **(kg/m^2^)	24.3 ± 3.7	25.1 ± 3.8	25.2 ± 5.5	.776	.557
**Waist-Hip index**	0.92 ± 0.09	0.92 ± 0.09	0.91 ± 0.11	.459	.557
**Whole-body muscle mass **(kg)	24.5 ± 6.4	25.7 ± 5.6	27.1 ± 9.2	.691	1
**Diagnoses **(number)	3.2 ± 1.0	3.1 ± 1.0	2.9 ± 0.8	.502	.725
**Medications **(number)	5.6 ± 1.7	5.8 ± 1.6	4.7 ± 1.7	.227	.164
**OA/B **^a ^(number)	10/3	7/3	8/3	-	-

^a^ADL-category according to Katz et al. [13]. Mann-Whitney U Test (exact 2-tailed significance) Control versus WBV+ ^b^initial randomisation ^c^reassessed at 6 weeks. Values represent number or mean ± SD.

**Table 3:** Participants' functional performance at baseline.

**Parameter**	**WBV+**		**Control N = 11**	**p^a^**	**p^b^**
				
	Initial Randomisation (N = 13)	Reassessed at 6 weeks (N = 10)			
**Chair sit-and-reach **(cm)	-20.2 ± 6.2	-21.0 ± 6.9	-23.2 ± 9.4	.061	.145
**Back scratch **(cm)	-23.2 ± 16.0	-23.0 ± 18.3	-15.9 ± 6.9	.323	.667
**30-second chair stand **(number)	6.3 ± 4.0	7.0 ± 4.1	8.2 ± 3.1	.127	.303
**Tinetti test**					
**Body balance **(score/16)	12.8 ± 3.7	13.4 ± 3.1	13.2 ± 2.6	.784	.566
**Gait **(score/12)	9.6 ± 2.7	9.9 ± 2.8	9.9 ± 2.1	.891	.822
**Total **(score/28)	22.4 ± 5.9	23.3 ± 5.6	23.1 ± 4.3	.966	.665
**Timed get-up-and-go test **(seconds)	17.9 ± 9.3	15.3 ± 5.5	14.8 ± 6.3	.399	.743
**Grip strength **(KPa)	41.6 ± 19.5	43.3 ± 18.9	43.3 ± 24.6	.765	.545
**Leg extension 40 cm/sec**					
**Work **(J)	66.9 ± 74.6	55.8 ± 44.6	88.5 ± 79.4	.361	.387
**Maximal force **(N)	270.0 ± 203.8	251.3 ± 141.4	375.2 ± 253.8	.277	.282
**Maximal power **(W)	108.0 ± 81.5	100.5 ± 56.5	150.1 ± 101.5	.277	.282
**Maximal explosivity **(N/sec)	2693.1 ± 1698.3	2755.0 ± 1600.1	4070.0 ± 2483.0	.134	.173
**Leg extension 60 cm/sec**					
**Work **(J)	47.1 ± 57.1	36.9 ± 32.9	68.7 ± 78.6	.459	.468
**Maximal force **(N)	204.3 ± 197. 0	178.3 ± 148.1	312.1 ± 281.3	.283	.290
**Maximal power **(W)	123.4 ± 117.4	108.0 ± 87.7	187.3 ± 168.7	.339	.359
**Maximal explosivity **(N/sec)	3885.0 ± 3291.6	3553.5 ± 2700.0	4872.3 ± 3371.6	.424	.426

Mann-Whitney U Test (exact 2-tailed significance) Control versus WBV+ ^a^initial randomisation ^b^reassessed at 6 weeks. Values represent mean ± SD.

**Table 4:** Change in functional performance.

**Parameter**	**WBV+ (N = 10)**				**Control (N = 11)**			**p^c^**	**p^d^**	**p^e^**
				
	**Baseline**	**6 weeks**	**p^a^**	**p^b^**	**Baseline**	**6 weeks**	**p^a^**			
**Chair sit-and-reach **(cm)	-21.0 ± 6.9	-18.1 ± 8.0	.031	.506	-23.2 ± 9.4	-19.9 ± 9.7	.113	1	.817	.486
**Back scratch **(cm)	-23.0 ± 18.3	-21.2 ± 18.4	.291	.989	-15.9 ± 6.9	-17.6 ± 8.2	.424	.178	.173	.618
**30-second chair stand **(number)	7.0 ± 4.1	8.8 ± 5.4	.062	.252	8.2 ± 3.1	8.3 ± 4.0	.844	.188	.200	.637
**Tinetti test**										
**Body Balance **(score/16)	13.4 ± 3.1	13.9 ± 2.5	.250	.938	13.2 ± 2.6	11.8 ± 3.1	.008	.001	.001	.031
**Gait **(score/12)	9.9 ± 2.8	9.5 ± 3.1	.125	.031	9.9 ± 2.1	9.5 ± 2.3	.063	1	.680	.700
**Total **(score/28)	23.3 ± 5.6	23.4 ± 5.5	1	.516	23.1 ± 4.3	21.3 ± 4.9	.004	.002	.001	.048
**Timed get-up & go **(seconds)	15.3 ± 5.5	12.0 ± 3.7	.008	.113	14.8 ± 6.3	14.3 ± 7.1	.492	.029	.075	.238
**Grip strength **(KPa)	43.3 ± 18.9	44.6 ± 20.8	.367	.904	43.3 ± 24.6	45.6 ± 25.4	.102	.973	.878	.562
**Leg extension 40 cm/sec**										
**Work **(J)	55.8 ± 44.6	92.7 ± 83.9	.006	.034	88.5 ± 79.4	119.3 ± 103.0	.007	.989	.661	.496
**Max. force **(N)	251.3 ± 141.4	397.6 ± 270.5	.020	.052	375.2 ± 253.8	482.4 ± 313.8	.032	.523	.797	.940
**Max. power **(W)	100.5 ± 56.5	158.1 ± 107.4	.020	.064	150.1 ± 101.5	193.0 ± 125.5	.032	.512	.773	.962
**Max. explosivity **(N/sec)	2755.0 ± 1600.1	4800 ± 4187.9	.020	.078	4070.0 ± 2483.0	5151.8 ± 2840.8	.123	.848	1	.640
**Leg extension 60 cm/sec**										
**Work **(J)	36.9 ± 32.9	62.3 ± 56.0	.020	.077	68.7 ± 78.6	88.4 ± 96.4	.005	.987	.576	.496
**Max. force **(N)	178.3 ± 148.1	342.8 ± 287.0	.004	.027	312.1 ± 281.3	398.9 ± 334.8	.005	.654	.962	.819
**Max. power **(W)	108.0 ± 87.7	205.7 ± 172.2	.010	.052	187.3 ± 168.7	239.3 ± 200.9	.005	.605	.915	.866
**Max. explosivity **(N/sec)	3553.5 ± 2700.0	6373.5 ± 5249.4	.010	.027	4872.3 ± 3371.6	7093.6 ± 5412.1	.019	.809	.867	.682

Wilcoxon Signed Ranks Test (exact 2-tailed significance): ^a^with (N = 12) & without (N = 10) Last Observation Carried Forward approach, ^b^Worst Rank Score analysis (N = 12); Mann-Whitney U Test (exact 2-tailed significance): ^c^N = 21, ^d ^Last Observation Carried Forward approach (N = 23), ^e^Worst Rank Score analysis (N = 23).

As can be seen in table 4, changes in performance on timed up-and-go and Tinetti-test (for body balance and total score) were significantly better for the WBV+ compared to the control group (p = 0.029 for timed up-and-go, p = 0.001 and p = 0.002 for Tinetti body balance and total score respectively). In fact, subjects of the WBV+ group improved significantly on the timed up-and-go test (p = 0.008), whereas no change was observed in the controls. Balance, as observed by the Tinetti-test, worsened significantly in the controls (p = 0.008 for Tinetti body balance and p = 0.004 for Tinetti total score) but remained unchanged in the WBV+ group. In the WRS analysis the difference in change on the Tintetti-test between the WBV+ and control group remained significant (p = 0.031 and p = 0.048 for Tinetti body balance and total score respectively); the difference in change on the timed get-up-and-go test was not significant anymore (p = 0.238). In the LOCF approach, the changes were in the same direction and remained significant, except for the difference in change on the timed up-and-go test with a p-value now slightly higher than the significance level (p = 0.075). Significance levels of changes within the WBV+ group remained the same for the LOCF approach (considered as ties in the Wilcoxon Signed Ranks Test).

As shown in table 4, significant improvements in leg extension performance were observed in both groups (all p < 0.05, except for explosivity at 40 cm/sec for the control group), and chair sit-and-reach improved significantly in the WBV+ group (p < 0.05); which were attenuated in the WRS analysis. However, no significant difference in change between both groups was observed for any of the approaches.

## Discussion

In this study we have investigated the feasibility and benefit of 6 weeks WBV in institutionalised elderly persons. The results of our study indicate that WBV might have beneficial effects on balance and mobility in elderly nursing home residents. Indeed, subjects assigned to WBV improved significantly on the timed up-and-go test and maintained their baseline level of balance (as measured by Tinetti-test), contrary to the controls who did not improve in timed up-and-go and who's balance worsened significantly (difference in change between both groups p = 0.029 for timed up-and-go, p = 0.001 and p = 0.002 for Tinetti body balance and total score respectively). It might be possible that the decrease of balance scores in the controls reflects the frail and physically unstable status of nursing home residents. The challenge for keeping equilibrium on the vibration platform in subjects of the WBV+ group could result in a possible adaptation mechanism that preserved them for further decline. Our results were in agreement with those from Bruyere et al. [25], who also found significant changes in timed up-and-go and Tinetti-test performance following WBV in institutionalised elderly. However, they used another type of WBV platform (Galileo, Orthometrix Inc., New-York) generating tilting oscillations; contrary to the Power-Plate, which produces vertical vibrations.

In our study, 3 subjects in the WBV+ group were lost for follow-up. One dropout was completely at random (airway infection), while two might have been related to the WBV program (one developed groin pain, the other one became afraid). This dropout rate corresponds to that reported in other WBV intervention studies [12,25,26] and classic intensive weight-lifting exercise [19] involving elderly subjects. It cannot be excluded that our results would have been different if these persons did not drop out [27]. For the latter two dropouts, a worst rank score analysis (substitution of missing end-evaluation values by the worst rank score, a technique used when subjects are lost for follow-up due to absorbing events like death [24]) was carried out. In a WRS analysis, the difference in change on the timed get-up-and-go test was not significant anymore (p = 0.238). However, during our repeated attempts to convince them to participate in the end-evaluations [23] we observed no visible worsening in their daily functioning, and we feel that WRS analysis is less appropriate here. Therefore we have substituted the missing end-evaluations of the two latter subjects by carrying the last available observation forward as an alternative procedure [25]. In this approach, the difference in change on the timed get-up-and-go test was attenuated and the p-value was slightly higher than the threshold-value for statistical significance (p = 0.075). LOCF assumes that the last observation of a subject who dropped out the study is an unbiased representation of what the missing value would have been had the subject been followed. It is obviously an untestable assumption, leading to distortion of the covariance structure of the data as well as the mean values, and LOCF should be interpreted with great caution [23].

After 6 weeks, both groups (WBV+ and control) showed significantly better leg extension performance compared to baseline (except for explosivity at 40 cm/sec for the control group, all p < 0.05). However, differences in change between both groups were not statistically significant. We observed a high variability for leg extension performance. None of the participants showed difficulties in performing the tests indicating that this variability reflected their heterogeneous condition. Important age-related variability, indeed, is a characteristic of all studies involving geriatric populations, especially when dealing with frail elderly persons. Moreover, our previous studies indicate that this test procedure is applicable in elderly persons [19,28]. The important improvements of the controls can be explained by the fact that these persons performed exactly the same exercises as the WBV+ group, except that the platform did not vibrate. Probably the low baseline muscle performance level of the participants predetermined the possibility to obtain considerable gains in a short time. These results indicate that frail elderly are highly trainable by means of simple physical exercise (i.e., maintaining weight-bearing positions).

In our study, we assumed that if WBV is effective, adaptations in muscle function would become measurable within short time (6 weeks). Moreover, prolongation over a longer period might result in occurrence of confounding factors in these frail nursing home residents (acute disease such as influenza, cognitive decline, changes in medication use and instability of comorbidity). However, it is not excluded that higher benefit of WBV on muscle performance might be obtained after longer or more intensive training programmes. Russo et al. [26] described significantly improved lower limb muscle power in elderly independently living women following 6 months WBV compared to control. Their study differed from ours since they used a tilting-platform (Galileo) and their control group performed no physical exercise at all. Roelants et al. [12] reported significantly improved knee extensor strength following 24 weeks WBV in community-dwelling elderly women. These improvements were similar to those obtained with traditional weight-lifting strength training. Also in their study the controls did not perform any exercise at all. Moreover, their WBV program consisted in much more intensive exercises (both static and dynamic, including one-legged) with a total training duration going up to 30 minutes. In our study, only bipodal static exercises were performed, with a maximal exercise duration attaining 5 to 6 minutes WBV (10 to 15 minutes including rest periods). One of the potential mechanisms leading to muscle adaptation following WBV is by stimulating the tonic vibration reflex. Possibly, longer and/or more intensive WBV exercise sessions might result in higher motor unit activation, and thus better training effects [10].

Lower body flexibility (as measured by the chair sit-and-reach test) improved significantly in the WBV+ group (p < 0.05, not in WRS analysis), but not in the control group. To our knowledge, this is the first paper describing improved flexibility in elderly persons following WBV. The training-induced change in flexibility, however, was not significantly different between WBV and control group. It is assumed that vibration can improve flexibility by central mechanisms such as increase in stretch tolerance (higher pain threshold) and the stimulation of Golgi tendon organs (contraction inhibition) [29]. Since the participants in our study firmly held the front handle of the vibration device, the vibration stimulus was also partly transmitted through the upper limbs. However, no significant changes in upper limb flexibility were observed.

The participants who completed the WBV+ program attended 96% of the exercise sessions, compared to 86% in the control group. These attendance rates correspond to those in common geriatric rehabilitation practice. It can, however, not be excluded that this small difference in compliance (10% = average of 1.8 sessions over 6 weeks) might have affected our results.

The high rate of compliance in our study (96% in the WBV+ group) supports the feasibility of WBV in frail institutionalised elderly. However, the majority (66%) of the nursing home residents at the moment of the study were excluded: 36 (36%) were not eligible and 29 (30%) presented exclusion criteria. Possibly, our inclusion and exclusion criteria were too severe, although it seems reasonable to assume that the risk for complications during WBV in subjects presenting severe levels of dependency or cognitive decline may be much higher than in the participants in our study. Therefore, the feasibility of WBV for these categories of frail institutionalised elderly remains further to be studied and it might be necessary to develop more adapted exercise programs.

## Conclusion

Overall we can conclude that 6 weeks static WBV exercises are feasible in elderly nursing home residents with limited functional dependency, and might be beneficial for balance and mobility. It is still insufficiently clear whether WBV has supplementary benefit on muscle performance and flexibility compared to classic exercise in these persons.

## Competing interests

The author(s) declare that they have no competing interests.

## Authors' contributions

TM and IB conceived the study. TM participated in the coordination of the study, the analysis and the redaction. IB performed the statistical analysis and the redaction, and participated in the coordination of the study, the supervision of the exercise sessions and the measurements. EVH participated in the supervision of the exercise sessions and the measurements. JCL participated in the coordination of the study and the evaluation of the health condition of the participants. All authors read and approved the final manuscript.

## Pre-publication history

The pre-publication history for this paper can be accessed here:



## References

[B1] Fried LP, Tangen CM, Walston J, Newman AB, Hirsch C, Gottdiener J, Seeman T, Tracy R, Kop WJ, Burke G, McBurnie MA (2001). Frailty in Older Adults: Evidence for a Phenotype. J Gerontol A Biol Sci Med Sci.

[B2] Bortz WM (2002). A Conceptual Framework of Frailty: A Review. J Gerontol A Biol Sci Med Sci.

[B3] Morley JE, Perry HM, Miller DK (2002). Editorial: Something about frailty. J Gerontol A Biol Sci Med Sci.

[B4] Latham NK, Bennett DA, Stretton CM, Anderson CS (2004). Systematic review of progressive resistance strength training in older adults. J Gerontol A Biol Sci Med Sci.

[B5] Rydwik E, Frandin K, Akner G (2004). Effects of physical training on physical performance in institutionalised elderly patients (70+) with multiple diagnoses. Age Ageing.

[B6] Thomas VS, Hageman PA (2003). Can neuromuscular strength and function in people with dementia be rehabilitated using resistance-exercise training? Results from a preliminary intervention study. J Gerontol A Biol Sci Med Sci.

[B7] Fiatarone MA, Marks EC, Ryan ND, Meredith CN, Lipsitz LA, Evans WJ (1990). High-intensity strength training in nonagenarians. Effects on skeletal muscle. Jama.

[B8] Fiatarone MA, O'Neill EF, Ryan ND, Clements KM, Solares GR, Nelson ME, Roberts SB, Kehayias JJ, Lipsitz LA, Evans WJ (1994). Exercise training and nutritional supplementation for physical frailty in very elderly people. N Engl J Med.

[B9] Liao S, Ferrell BA (2000). Fatigue in an older population. J Am Geriatr Soc.

[B10] Delecluse C, Roelants M, Verschueren S (2003). Strength increase after whole-body vibration compared with resistance training. Med Sci Sports Exerc.

[B11] de Ruiter CJ, Van Raak SM, Schilperoort JV, Hollander AP, de Haan A (2003). The effects of 11 weeks whole body vibration training on jump height, contractile properties and activation of human knee extensors. Eur J Appl Physiol.

[B12] Roelants M, Delecluse C, Verschueren SM (2004). Whole-Body-Vibration Training Increases Knee-Extension Strength and Speed of Movement in Older Women. J Am Geriatr Soc.

[B13] Katz S, Ford AB, Moskowitz RW, Jackson BA, Jaffe MW (1963). Studies of Illness in the Aged. The Index of ADL: A Standardized Measure of Biological and Psychosocial Function. Jama.

[B14] Berger VW, Weinstein S (2004). Ensuring the comparability of comparison groups: is randomization enough?. Control Clin Trials.

[B15] Bautmans I, Mets T (2005). A fatigue resistance test for elderly persons based upon grip strength: reliability and comparison with healthy young subjects. Aging Clin Exp Res.

[B16] Podsiadlo D, Richardson S (1991). The timed "Up & Go": a test of basic functional mobility for frail elderly persons. J Am Geriatr Soc.

[B17] Tinetti ME (1986). Performance-oriented assessment of mobility problems in elderly patients. J Am Geriatr Soc.

[B18] Rikli RE, Jones CJ (1999). Development and validation of a functional fitness test for community-residing older adults. Journal of Aging and Physical Activity.

[B19] Bautmans I, Njemini R, Vasseur S, Chabert H, Demanet C, Mets T (2005). Biochemical changes in response to intensive resistance exercise training in the elderly. Gerontology.

[B20] Lenaerts A, Verbruggen LA, Duquet W (2001). Reproducibility and reliability of measurements using a linear isokinetic dynamometer, Aristokin. J Sports Med Phys Fitness.

[B21] Bautmans I, Njemini R, Lambert M, Demanet C, Mets T (2005). Circulating Acute Phase Mediators and Skeletal Muscle Performance in Hospitalized Geriatric Patients. J Gerontol A Biol Sci Med Sci.

[B22] Martin AD, Spenst LF, Drinkwater DT, Clarys JP (1990). Anthropometric estimation of muscle mass in men. Med Sci Sports Exerc.

[B23] Lachin JM (2000). Statistical considerations in the intent-to-treat principle. Control Clin Trials.

[B24] Lachin JM (1999). Worst-rank score analysis with informatively missing observations in clinical trials. Control Clin Trials.

[B25] Bruyere O, Wuidart MA, Di Palma E, Gourlay M, Ethgen O, Richy F, Reginster JY (2005). Controlled whole body vibration to decrease fall risk and improve health-related quality of life of nursing home residents. Arch Phys Med Rehabil.

[B26] Russo CR, Lauretani F, Bandinelli S, Bartali B, Cavazzini C, Guralnik JM, Ferrucci L (2003). High-frequency vibration training increases muscle power in postmenopausal women. Arch Phys Med Rehabil.

[B27] Berger VW, Bears JD (2003). When can a clinical trial be called 'randomized'?. Vaccine.

[B28] Verbruggen LA, Lenaerts A, Duquet W, Pauwels C, Mets T (2000). Relationship between muscle strength in functional movements, activities of daily living and bone mineral density in osteoporotic women. Osteoporosis International.

[B29] Issurin VB, Liebermann DG, Tenenbaum G (1994). Effect of vibratory stimulation training on maximal force and flexibility. J Sports Sci.

[B30] Power-Plate. http://www.power-plate.us.

